# Diet, liver function and dimethylhydrazine-induced gastrointestinal tumours in Wistar rats

**DOI:** 10.1038/bjc.1979.127

**Published:** 1979-06

**Authors:** W. M. Castleden, K. B. Shilkin

## Abstract

**Images:**


Br. J. Cancer (1979) 39, 731

### DIET, LIVER FUNCTION AND DIMETHYLHYDRAZINE-INDUCED GASTROINTESTINAL TUMOURS IN MALE WISTAR RATS

W. M. CASTLEDEN* AND K. B. SHILKIN†

*From the *University of Western Australia, Queen Elizabeth II Medical Centre, and the †Department of Histopathology, Sir Charles Gairdner Hospital, Queen Elizabeth II Medical Centre, Nedlands, Western Australia 6009*

Received 6 October 1978 Accepted 8 February 1979

**Summary.—Male Wistar rats fed a normal laboratory pelleted diet, when treated s.c. with 1,2-dimethylhydrazine (DMH) 10 mg/kg/wk survived the 24-week experiment, showed no signs of chemical toxicity or macroscopic liver damage, and developed mainly large-bowel tumours. Conversely, male Wistar rats treated with 20 mg/kg/wk DMH did not survive the full term of the experiment and developed ascites, pleural effusions and nodular livers. They also developed more small-bowel tumours than large-bowel tumours. The relationship between the predominant site of tumour development and dosage of DMH was highly significant.**

**Male Wistar rats fed with an all-liquid diet (Vivonex) and treated with 20 mg/kg/wk DMH behaved quite differently both in terms of survival and site of tumour development. These rats survived the full term of the experiment, showed no signs of chemical toxicity, experienced minimal liver damage and developed predominantly large-bowel tumours. The protection afforded by the all-liquid diet against DMH toxicity and small-bowel tumour induction was statistically highly significant.**

**A series of blood tests with special reference to liver function confirmed the highly significant degree of protection against liver damage afforded by the all-liquid diet.**

**Sections of liver from treated rats were examined, and a simple pathological scoring system was devised which showed a highly significant difference in liver histology between standard diet and liquid-diet rats treated with 20 mg/kg/wk DMH.**

**The results strongly suggest an association between severity of liver damage from DMH and the subsequent development of small-bowel tumours. The all-liquid diet protected rats from liver damage and these rats developed significantly fewer small-bowel tumours.**

SINCE 1962, when naturally occurring cycasin from the cycad plant was found to be carcinogenic in laboratory rodents (Laqueur & Spatz, 1968), an exponential number of publications has appeared describing various experiments using cycasin or its metabolic analogues: 1,2-dimethylhydrazine (DMH), azoxymethane (AOM) and methylazoxymethanol (MAM). We have reviewed 4 papers published between 1967 and 1970; 14 between 1971 and 1973; 34 between 1974 and 1976; and ~50 which have appeared more recently. These fall into 4 main groups: (1) early studies of the gastrointestinal carcinogenicity of these compounds; (2) histopathology and tumour morphogenesis; (3) aetiological experiments; and (4) more specific carcinogenesis studies at the cellular level.

In general, it would seem that almost any alteration in the diet, or operative procedure, can alter the incidence of gastrointestinal tumours in rats treated with these compounds. Different species of rats have varying susceptibilities to gastrointestinal carcinogenesis with cycad analogues, and thus possible mechanisms of carcinogenesis cannot be deduced from the aetiological studies reported to date.

Some observations have been made on the morphological changes in the liver cells in animals treated with DMH (Ward *et al.*, 1973; Martin *et al.*, 1973; Hawks *et al.*, 1974) but a correlation of the degree of liver damage with the site of gastrointestinal cancers has received scant attention.

Earlier papers evaluating the results of experiments using cycasin or its analogues (Laqueur & Spatz, 1968; Weisberger, 1971) suggested that a carcinogenic metabolite might be delivered *via* the bile to the gastrointestinal tract. More recently this has been refuted by other workers (Hawks & Magee, 1974; Fiala, 1975; 1977) who have used $^{14}C$-labelled DMH, and have recovered only small amounts of the isotope from the rat bile. Hence there is a need for clarification of the role of the liver in the carcinogenesis model. In a preliminary report (Castleden, 1977) it was shown that at necropsy the livers of rats treated with DMH at a dose of 20 mg/kg/wk appeared "granular", whereas the livers of rats treated with 10 mg/kg/wk DMH appeared macroscopically normal. In that report the 20 mg/kg/wk DMH-treated rats died early in the experiment from chemical toxicity. Here we describe these changes in more detail, and give the results of a further experiment designed to investigate the relationship between hepatocellular injury and gastrointestinal tumour development, comparing the effect of the all-liquid diet with the standard diet in male Wistar rats treated with DMH.

### MATERIALS AND METHODS

Male Wistar rats, bred at the Animal Breeding Centre of the University of Western Australia, 8–10 weeks old and weighing ~230 g, were used in these experiments. They were weighed and injected s.c. at the same time each week with 2% (w/v) 1,2-dimethylhydrazine dihydrochloride (Aldrich Chemical Co. Inc.) in normal saline containing 1·5% (w/v) EDTA, pH 6·4, at dosages of either 10 or 20 mg DMH base/kg body wt. Dosages of 20 mg/kg/wk DMH were administered for 20 weeks. Dosages of 10 mg/kg/wk DMH were administered for 22 weeks. Control rats were injected weekly with an equivalent volume of normal saline containing 1·5% (w/v) EDTA, pH 6·4. Injections were given beneath the lax skin of the groin region, the sides being alternated from week to week. All surviving rats were killed 2 weeks after their last injection.

Blood (8–10 ml) was obtained by heart puncture after ether anaesthesia. Liver function tests (bilirubin, alkaline phosphatase, aspartic serum transaminase (SGOT), serum albumin and total protein levels) were measured using a Vickers MC 300 Multichannel Auto Analyser.

Full necropsies were carried out on all except 10 of the 246 animals. These 10 rats, treated with 20 mg/kg/wk DHM, died unexpectedly early in the experiment and were either partly cannibalized or autolysed. Subsequently, moribund animals were killed with ether and necropsy undertaken immediately. All areas of nodularity in the gastrointestinal tract were measured and indicated on specially designed charts, and appropriate gut and liver tissue was fixed in 10% buffered formol saline. All sections taken from paraffin blocks were stained with haematoxylin and eosin, and representative sections were stained by PAS before and after diastase digestion. Some formalin-fixed liver was stained, after frozen section, with Oil-Red-O to demonstrate lipid.

Standard sections of liver from 77 rats were examined by one of us (K.B.S.) without knowledge of the dosage of DMH or diet used. Central and peripheral zones of the hepatic lobule were studied and separately scored from 0 to 3 on arbitrary scales for the degree of (1) liver-cell degeneration, (2) hepatocytic necrosis and (3) inflammatory infiltration. However, there were no statistically significant differences between changes in the central and peripheral zones, and these two scores were pooled. Total scores for each group were calculated as a percentage of the total possible score.

Two experiments were conducted. Rats in the first experiment were fed 1 of 7 diets, the details of which have been previously reported (Castleden, 1977). Essentially Diets 1 to 6 consisted of Milne's standard laboratory diet with or without the addition of various bulking agents. There was no significant difference in tumour incidence with any of these solid diets, which have therefore been grouped together (see Table I) as "Solid Diets". In the second experiment, rats were fed either Milne's standard laboratory diet and treated with 20 mg/kg/wk DMH, or they were fed the all-liquid diet and injected with either DMH solute alone or with 20 mg/kg/wk DMH. The standard diet was fed *ad libitum*, whilst the all-liquid diet was administered 12-hourly because of possible deterioration if kept for longer periods at room temperature.

TABLE I.—*Number of rats in each dietary group*

| | Control (No DMH) | 10 mg/kg/wk DMH | 20 mg/kg/wk DMH |
|---|---|---|---|
| Solid diets[1] | | | |
| Exp. 1 | 30 | 60 | 93 |
| Exp. 2 | — | — | 20 |
| Total | 30 | 60 | 113 |
| All-liquid diet[2] (VivonexR) | | | |
| Exp. 1 | 5 | 8 | 10 |
| Exp. 2 | 5 | — | 15 |
| Total | 10 | 8 | 25 |

Notes:
[1] Milne's laboratory diet alone or separately supplemented (see Castleden, 1977). Milne's laboratory diet is a standardized, pelleted, rodent diet made from a mixture of cereals, fish meal, milk powder, sugar, tallow and yeast, yielding on analysis 21·2% crude protein (3·3% nitrogen), 4·9% crude fat, 4·4% crude fibre, 5·3% ash, 11·5% moisture and 52·7% nitrogen-free extractives (carbohydrate), with standard concentrations of essential minerals, including 1·01% Ca, 0·16% Mg, 0·29% Na, 0·78% K, 38 parts/$10^6$ Mn, 170 parts/$10^6$ Cu, 230 parts/$10^6$ Fe, 160 parts/$10^6$ Zn, 7% $PO_4$ (as P).
[2] Vivonex® (Norwich Eaton) 160 g (2 sachets dissolved in 900 ml water) which was administered to rats in the liquid-diet group twice daily; these rats received no pellets. This diet contains 1·22% N as pure L-amino acids, 0·54% fat (safflower oil) and 85% carbohydrate with essential vitamins and minerals, including 1·66% Ca, 0·72% Mg, 3·22% Na, 4·38% K, 58·4 parts/$10^6$ Mn, 40·5 parts/$10^6$ Cu, 207·5 parts/$10^6$ Fe, 8·37 parts/$10^6$ Zn, 1·66% $PO_4$ (as P).

Table I shows the number of rats in each group with further details of the composition of the two dietary groups.

In the first experiment, the liquid-diet rats lost weight for the first 8–10 days after transition from their previous standard diet. Thereafter weekly weight was the same for both dietary groups. In the second experiment there was comparable weekly weight gain in both the liquid-diet and standard-diet animals. Both diets were therefore presumed to be isocaloric.

### RESULTS

### *Survival*

*Control group.*—All 40 rats in this group survived the whole 24 weeks of the experiment without any clinical or histological evidence of abnormality.

*10mg/kg/wk group.*—In this group, 62 of the 68 rats treated with weekly injections of 10 mg/kg/wk DMH survived until the 24th week of the experiment (after a total dose of 220 mg/kg DMH). They were then killed. Six solid-diet rats were killed shortly before the end of the experiment because of clinical evidence of tumour development, as judged by anaemia or bleeding from the rectum or ear. There was no clinical or gross necropsy evidence of chemical toxicity in any of the 10mg/kg/wk rats.

*20mg/kg/wk group.*—The survival time of the 20mg/kg/wk rats in the first experiment has already been reported (Castleden, 1977). Similar results were obtained in the second experiment, in which all 20 rats on the standard diet died early, presumably from chemical toxicity, by the 18th week of injections (Fig. 1). Once again the rats on the all-liquid diet treated with 20 mg/kg/wk DMH survived the full term of the experiment, with the exception of one rat which was killed in the 19th week of the experiment because of rectal bleeding. Necropsy revealed that the bleeding was due to the intussusception of a small-bowel tumour. The mean survival of the 20 standard-diet rats in the second experiment was $112 \pm 1·9$ days, compared to $153 \pm 1·0$ days for the 15 liquid-diet rats.

### *Tumour analysis*

A detailed tumour analysis of the first group of experiments has already been published (Castleden, 1977). Fig. 2 shows the site of tumour development in the animals receiving 10 mg/kg/wk DMH and Fig. 3 shows the same for animals with 20 mg/kg/wk DMH. In the first dietary experiment 60 solid-diet rats were treated with 10 mg/kg/wk DMH and these developed 29 small-bowel tumours and 85

FIG. 1.—Survival curve for second dietary experiment. 20 standard-diet rats (+) were all dead by the 18th week of the experiment, when 15 liquid-diet rats (▼) were still alive. All rats received 20 mg/kg/wk DMH.

FIG. 2.—Necropsy chart showing the sites of tumour development in rats treated with 10 mg/kg/wk DMH in the first dietary experiment.

FIG. 3.—Necropsy chart showing the site of tumour development in rats treated with 20 mg/kg/wk DMH in the first dietary experiment.

TABLE II.—*First dietary experiment: total number of tumours in all groups on solid diet*

| | 10 mg/kg/wk DMH (60 rats) | 20 mg/kg/wk DMH (75 rats*) |
|---|---|---|
| Small bowel | 29 | 84 |
| Large bowel | 85 | 60 |

$\chi^2 = 27{\cdot}97, P = < 0{\cdot}001$

* Rats dying before the 13th week of the experiment were excluded: no tumours were observed in any dietary group before this.

large-bowel tumours. Of the solid-diet rats, 75 survived 13 or more weeks of injections with 20 mg/kg/wk DMH, and they developed 84 small-bowel tumours and 60 large-bowel tumours. Table II summarizes these results. The higher dose of DMH not only caused early death from presumed chemical toxicity but also caused a highly significant increase in small-bowel tumours ($\chi^2 = 27{\cdot}97$, $P < 0{\cdot}001$). Tumour development in the second experiment correlated very closely with the experience with similar animals in the first group of experiments. This is shown in Table III. In both experiments the liquid-diet animals were clinically protected from chemical toxicity and developed significantly fewer small-bowel tumours than

TABLE III.—*Standard-diet and liquid-diet survival and tumour incidence in 20 mg/kg/wk DMH-treated rats in both experiments, showing close reproducibility of results*

| | Standard diet | | | | | | Liquid diet | | | | | |
|---|---|---|---|---|---|---|---|---|---|---|---|---|
| | No. of rats | | | Mean survival | Small-bowel tumours | Large-bowel tumours | No. of rats | | | Mean survival | Small-bowel tumours | Large-bowel tumours |
| | (a) | (b) | (c) | (days) | | | (a) | (b) | (c) | (days) | | |
| 1st Experiment | 20 | 12 | 8 | 104 | 9 | 4 | 10 | 10 | 8 | 155 | 1 | 11 |
| 2nd Experiment | 20 | 16 | 13 | 112 | 14 | 10 | 15 | 15 | 12 | 153 | 4 | 15 |
| Totals | 40 | 28 | 21 | 109 | 23 | 14 | 25 | 25 | 20 | 154 | 5 | 26 |

(a) Treated.

(b) Surviving to 13 weeks when first tumour-bearing rat was identified.

(c) With tumours at necropsy.

TABLE IV.—*Distribution of gastrointestinal tumours in tumour-bearing rats treated with 20 mg/kg/wk DMH and fed either standard or all-liquid diet*

| | Standard diet (21 rats) | All-liquid diet (20 rats) |
|---|---|---|
| Small-bowel tumours | 23 | 5 |
| Large-bowel tumours | 14 | 26 |
| | (mean total dose—310 mg/kg DMH) | (mean total dose—400 mg/kg DMH) |

$\chi^2 = 14{\cdot}76, P < 0{\cdot}001$

FIG. 4.—Changes in liver-function tests showing percentage deviation from control levels in rats on either standard diet or liquid diet, treated with 2 doses of DMH. ○ bilirubin; △ alkaline phosphatase; □ aspartic serum transaminase (SGOT); ▽ albumin; ◇ total protein. A control; B 10 mg/kg/wk DMH; C 20 mg/kg/wk DMH.

the standard-diet animals, which died early ($\chi^2 = 14{\cdot}76$, $P < 0{\cdot}001$: see Table IV).

*Blood tests*

Blood was obtained from 29 standard-diet and 36 liquid-diet rats. The results are shown in Fig. 4, where the median percentage deviation from control values is shown for 5 different liver function tests. The greatest deviations were observed in the standard-diet rats receiving 20 mg/kg/wk DMH. Standard univariate and multivariate analyses were performed on the complete blood data. The deviations observed in the all-liquid diet rats receiving 20 mg/kg/wk DMH, and in both dietary groups receiving 10 mg/kg/wk DMH were significantly lower, indicating much less liver damage.

*Liver histology*

The results of histological examination and scoring are shown in Table V.

In general, because of the clear-cut

TABLE V.—*Pathology scores† of liver damage in DMH-treated rats on two different diets*

| Group (No.) | Degeneration | Necrosis | Inflammation |
|---|---|---|---|
| 1. Control (15) | 13·3 | 0·0 | 0·0 |
| 2. Liquid diet (25) 20 mg/kg/wk DMH | 68·6* | 0·0 | 16·7* |
| 3. Standard diet (37) 20 mg/kg/wk DMH | 75·6 | 47·2** | 56·3** |

† Percentage of the maximum possible score.

* Group 2 significantly different from Group 1 ($P < 0{\cdot}001$).

** Group 3 significantly different from Group 2 ($P < 0{\cdot}001$).

FIG. 5.—*Control*—Liver from an animal which had not received DMH and which had been on an all-liquid diet. The histological appearances of the liver are essentially normal, apart from some minimal cytoplasmic vacuolation. H. & E. ×490.

FIG. 6.—*20 mg/kg/wk DMH and all-liquid diet*—Liver from an animal that was scored as 3 for vacuolation and 0 for necrosis and inflammation. Note the severe degree of vacuolation of virtually all hepatocytes and the absence of necrosis or inflammatory infiltrate. H. & E. ×490.

FIG. 7.—20 *mg/kg/wk DMH and standard diet*—Liver from an animal that was scored 2 for vacuolation, 3 for necrosis and 3 for inflammation. A number of vacuolated cells are apparent, but the presence of necrosis is more obvious and there is a fairly intense inflammatory infiltrate. H. & E. ×490.

differences in the degree of liver-cell vacuolation and/or inflammation, it was immediately obvious which liver sections had come from 20 mg/kg/wk DMH-treated rats (Figs. 6 & 7) and which had come from control rats (Fig. 5). These differences were compared using Student's *t* test: $t_{38} = 9{\cdot}32$, $P < 0{\cdot}001$ for control rats *vs* liquid-diet 20 mg/kg/wk DMH-treated rats; and $t_{50} = 12{\cdot}24$, $P < 0{\cdot}001$ for control rats *vs* standard diet 20 mg/kg/wk DMH-treated rats. Furthermore, there was no difficulty in determining which of the 20mg/kg/wk animals had been taking the all-liquid diet (Fig. 6) and which had been taking the standard diet (Fig. 7). There were obvious differences in the amount of cell necrosis and degree of inflammatory cell infiltrate ($t_{60} = 7{\cdot}93$, $P < 0{\cdot}001$), both of which were greater in the latter.

A more detailed account of these histological changes is being underaken.

### DISCUSSION

The first experiment showed that alteration in the diet affected survival as well as gastrointestinal tumour production when Wistar rats were treated with 20 mg/kg/wk DMH. An all-liquid elemental diet (Vivonex) enabled rats to survive 20 weeks of injections with 20 mg/kg/wk DMH, which they failed to do on a standard laboratory pelleted diet. The second experiment confirmed these observations.

All the rats, on either diet, survived repeated injections of 10 mg/kg/wk DMH. However, with this regime the predominant site of tumour development was different from that of rats treated with 20 mg/kg/wk DMH, which were significantly more likely to develop small-bowel tumours. This pattern of tumour distribution has been proposed as likely by Wiebecke *et al.* (1973) using DMH, and by Ward *et al.* (1973) using two different doses of AOM, but these workers had based their predictions on very small numbers of animals.

In both of our experiments there was a change of tumour distribution, with a significant reduction in the number of small-bowel tumours, in 20mg/kg/wk rats on the all-liquid diet. The apparent increase in large-bowel tumours in rats on the all-liquid diet may simply reflect their longer survival and their higher total dose of DMH (see Table IV). Blood tests demonstrated a high degree of liver protection by the all-liquid diet when rats were treated with 20 mg/kg/wk DMH, and also confirmed that standard-diet rats treated with 10 mg/kg/wk DMH suffered only small alterations in liver function.

There was histological evidence of severe hepatic toxicity in the standard-diet rats treated with 20 mg/kg/wk DMH, whereas feeding the all-liquid diet produced much less severe liver damage, both biochemically and morphologically.

Ten mg/kg/wk DMH is a carcinogenic dose for the strain of male Wistar rats we have used, inducing predominantly large-bowel tumours (Table II) and only slight liver damage. Twenty mg/kg/wk DMH is an excessive dose for these same animals when used as a model for colonic-cancer studies, since this dose increased the induction of small bowel tumours. The shift in tumour-site specificity with increased dose is an interesting phenomenon, and may provide a basis for a better understanding of the mechanism of action of this carcinogen. Equally important is the fact that a different diet, the Vivonex all-liquid diet, has the effect of negating the toxicity, liver damage and alteration in tumour site normally produced by the higher dose of DMH. The reason for this is not clear.

It is well established that many chemical carcinogens are metabolized to more proximate carcinogens in the liver (Heidelberger, 1977) and there is evidence that DMH is metabolized in the same manner (Shank & Magee, 1967; Hawks & Magee, 1974). The evidence presented here indicates that higher doses of DMH cause liver damage sufficient to impair normal hepatic function. Thus the rat-liver metabolism of DMH may be changed during the course of an experiment in which DMH is administered by weekly injections. Such changes could affect both the quantitative and qualitative distribution of metabolites, causing the altered profile of tumour distribution which we have observed in these experiments.

One possibility which we are further investigating is increasing biliary excretion of DMH and/or one or more of its carcinogenic metabolites as the liver becomes increasingly damaged. Isotope experiments (Hawks & Magee, 1974; Fiala, 1975) have shown that only small quantities ($<1\%$) of an injected dose of $^{14}C$-labelled DMH appear in the bile of rats with biliary fistulae. These experiments were of short duration and appeared to involve rats which had not previously been subjected to weekly injections of DMH. To our knowledge, no information is available on the biliary excretion of DMH and its metabolites in the later stages of tumour induction by weekly DMH injections. Furthermore, the importance of biliary excretion, whatever proportion of an injected dose this represents, remains to be established. This is of particular interest since, if any enterohepatic circulation of DMH and/or its metabolites were demonstrated, the results of many apparently conflicting animal metabolic, dietary and operative experiments could be explained.

The results of our experiments suggest that the liquid Vivonex diet in some way protects the rat liver from the toxic effects of the higher DMH dose. The mechanism of this protection may have important implications for further investigation of colonic carcinogenesis, and warrants further study.

There is increasing support for the concept that carcinoma of the colon in humans is probably caused, in part, by one or more environmental carcinogens (Vitale, 1975; Pratt *et al.*, 1977; Hill *et al.*, 1978). Possible pathways by which food products might be converted to DMH or its metabolites in humans have even been postulated (Fiala, 1977).

It is unwise to extrapolate from animal experiments to humans. Nevertheless, if chemical carcinogens are eventually proved to be implicated in human coloniccancer production, the enterohepatic circulation of such carcinogens could explain the differences in dietary (Burkitt, 1973; Wynder, 1975) and metabolic (Hill, 1975) epidemiology in humans.

### APPENDIX

Confirmation of the protective effect of Vivonex all-liquid diet in the rat DMH model has recently been presented to the British Society of Gastroenterology September 1978 meeting (Cruse *et al.*, 1978).

These experiments could not have been carried out without the support and assistance of the other members of the Department of Surgery in the University of Western Australia. Particular thanks are due to Dr Peter Detchon, Mrs Sue Kelly and Mrs Vicky Wakelam. We would also like to thank Mr B. Murphy for statistical advice, Mr H. Upenicks for help with the illustrations, Mr H. Whyte for technical help and Mrs Sheila Saville and Shirley Ann Poulton for typing.

The Vivonex used in these experiments was kindly supplied by Norwich-Eaton Laboratories of New York *via* Fawns and McAllan Pty Ltd of Melbourne.

### REFERENCES

BURKITT, D. P. (1973) Some diseases characteristic of modern western civilisation. *Br. Med. J.*, i, 274.

CASTLEDEN, W. M. (1977) Prolonged survival and decrease in intestinal tumours in dimethylhydrazine-treated rats fed a chemically defined diet. *Br. J. Cancer*, **35**, 491.

CRUSE, J. P., LEWIN, M. R., FERULANO, G. P. & CLARK, C. G. (1978) Dietary fibre, Vivonex, cholesterol and experimental colon cancer. *Gut*, **19**, A983.

FIALA, E. S. (1975) Investigations into the metabolism and mode of action of the colon carcinogen 1,2-dimethylhydrazine. *Cancer*, **36**, 2407.

FIALA, E. S. (1977) Investigations into the metabolism and mode of action of the colon carcinogens 1,2-dimethylhydrazine and azoxymethane. *Cancer*, **40**, 2436.

HAWKS, A. & MAGEE, P. N. (1974) The alkylation of nucleic acids of rat and mouse *in vivo* by the carcinogen 1,2-dimethylhydrazine. *Br. J. Cancer*, **30**, 440.

HAWKS, A., HICKS, R. M., HOLSMAN, J. W. & MAGEE, P. N. (1974) Morphological and biochemical effects of 1,2-dimethylhydrazine and 1-methylhydrazine in rats and mice. *Br. J. Cancer*, **30**, 429.

HEIDELBERGER, C. (1977) Chemical carcinogenesis. *Cancer*, **40**, 430.

HILL, M. J. (1975) Metabolic epidemiology of dietary factors in large bowel cancer. *Cancer Res.*, **35**, 3398.

HILL, M. J., MORSON, B. C. & BUSSEY, H. J. R. (1978) Aetiology of adenoma-carcinoma sequence in large bowel. *Lancet*, i, 245.

LAQUEUR, G. L. & SPATZ, M. (1968) Toxicology of cycasin. *Cancer Res.*, **28**, 2262.

MARTIN, M. S., MARTIN, F., MICHIELS, R. & 4 others (1973) An experimental model for cancer of the colon and rectum. *Digestion*, **8**, 22.

PRATT, C. B., RIVERA, G., SHANKS, E. & 4 others (1977) Colorectal carcinoma in adolescents: Implications regarding etiology. *Cancer*, **40**, 2464.

SHANK, R. C. & MAGEE, P. N. (1967) Similarities between the biochemical actions of cycasin and dimethylnitrosamine. *Biochem. J.*, **105**, 521.

VITALE, J. J. (1975) Possible role of nutrients in neoplasia. *Cancer Res.*, **35**, 3320.

WARD, J. M., YAMAMOTO, R. S. & BROWN, C. A. (1973) Pathology of intestinal neoplasms and other lesions in rats exposed to azoxymethane. *J. Natl Cancer Inst.*, **51**, 1029.

WEISBURGER, J. H. (1971) Colon carcinogens: their metabolism and mode of action. *Cancer*, **28**, 60.

WIEBECKE, B., KREY, U., LOHRS, U. & EDER, M. (1973) Morphological and autoradiographical investigations on experimental carcinogensis and polyp, development in the intestinal tract of rats and mice. *Virchows Arch.* (*Pathol. Anat.*), **360**, 179.

WYNDER, E. L. (1975) The epidemiology of large bowel cancer. *Cancer Res.*, **35**, 3388.


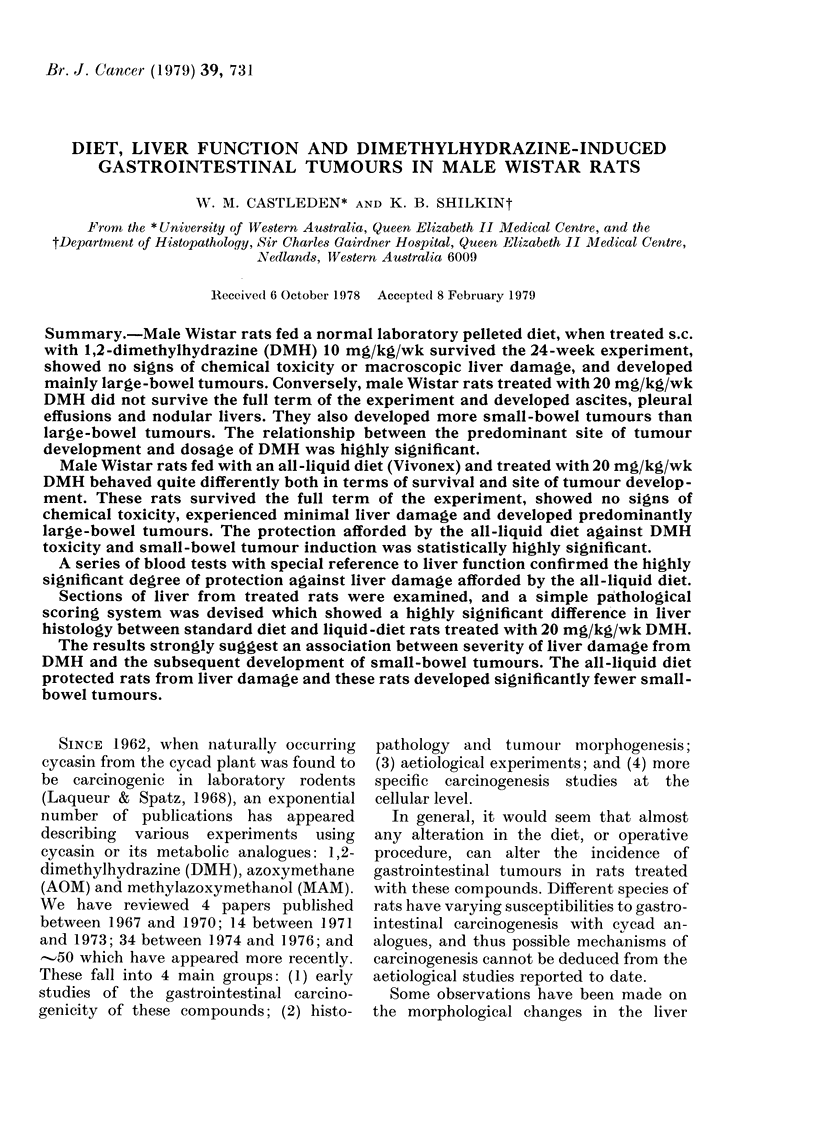

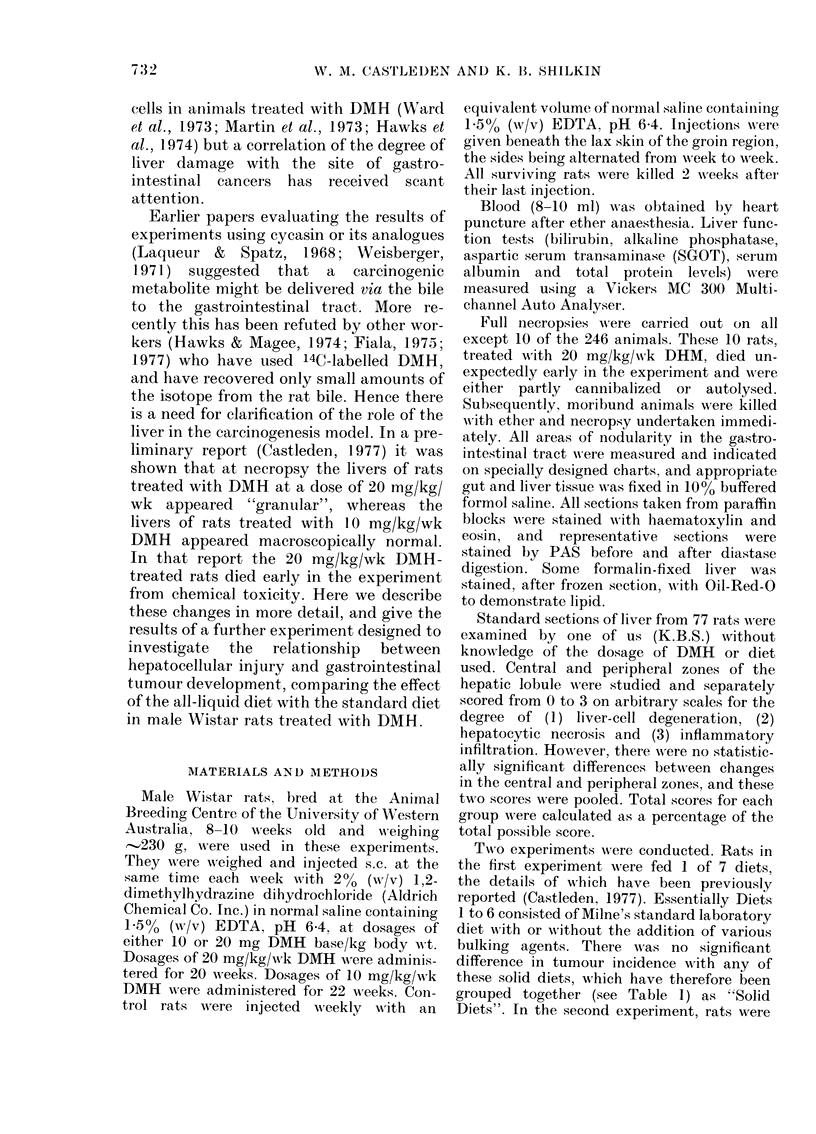

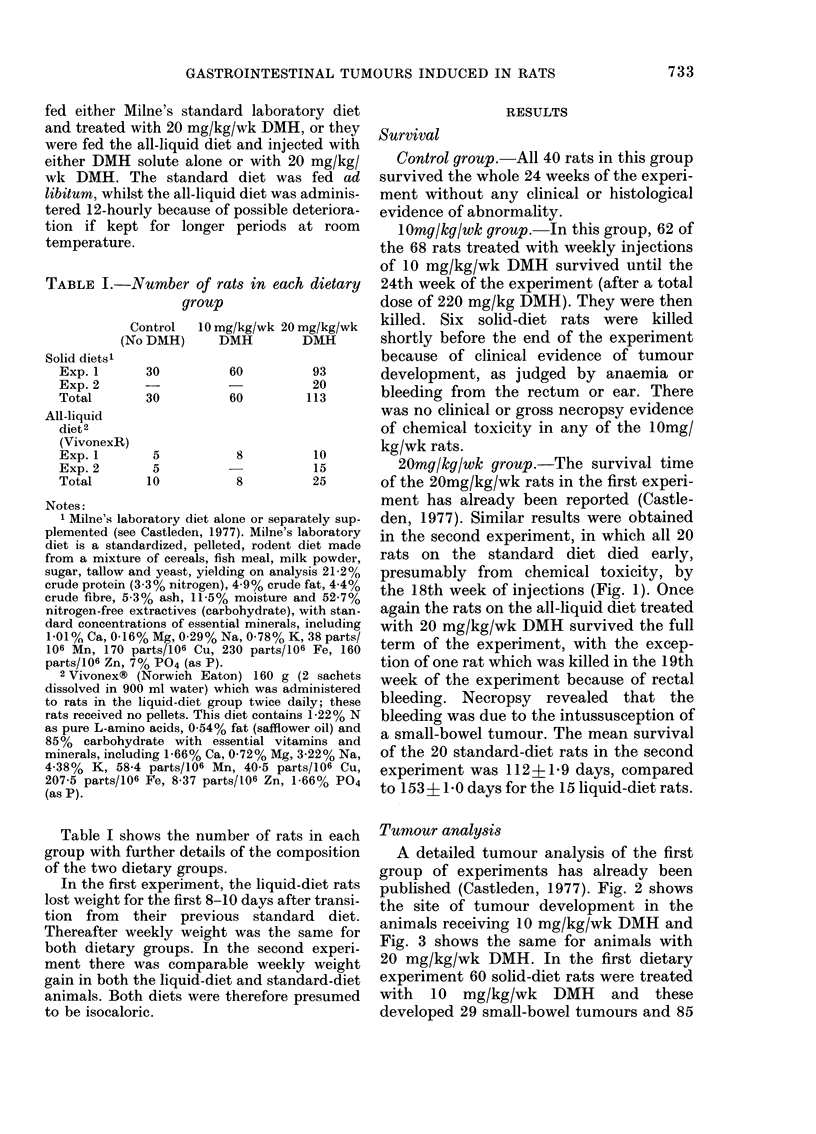

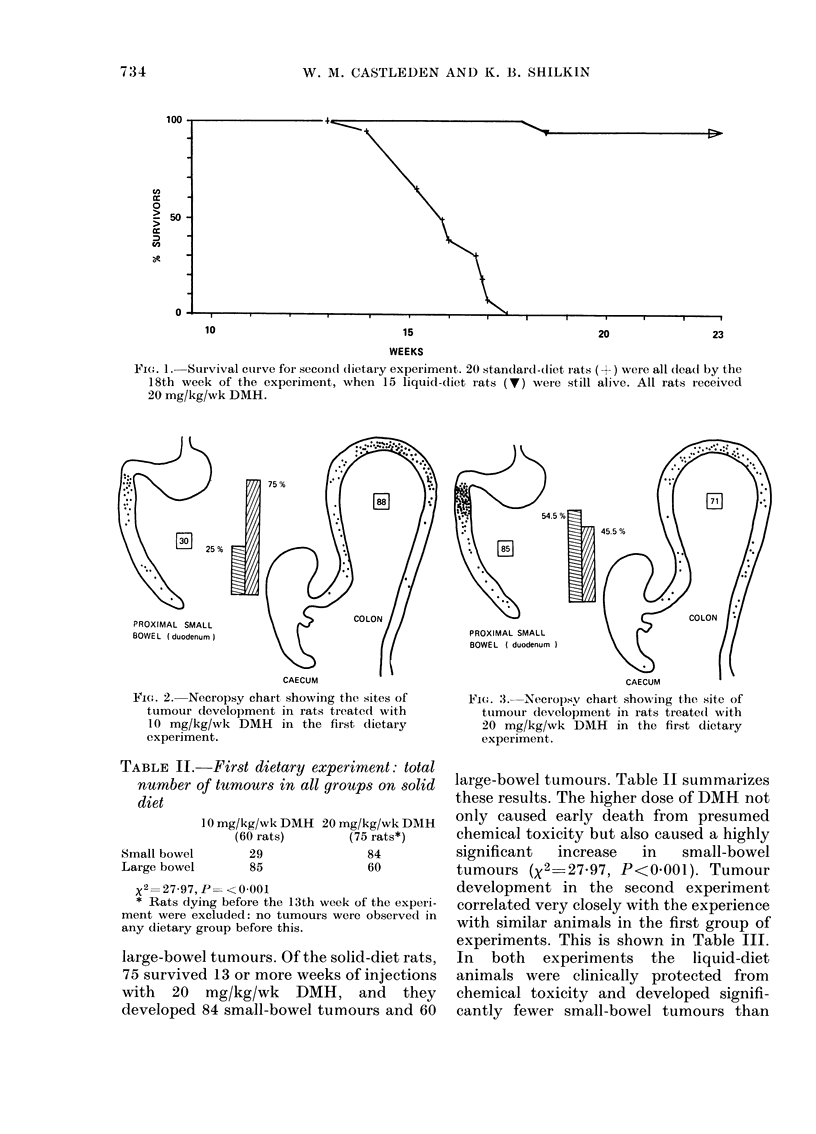

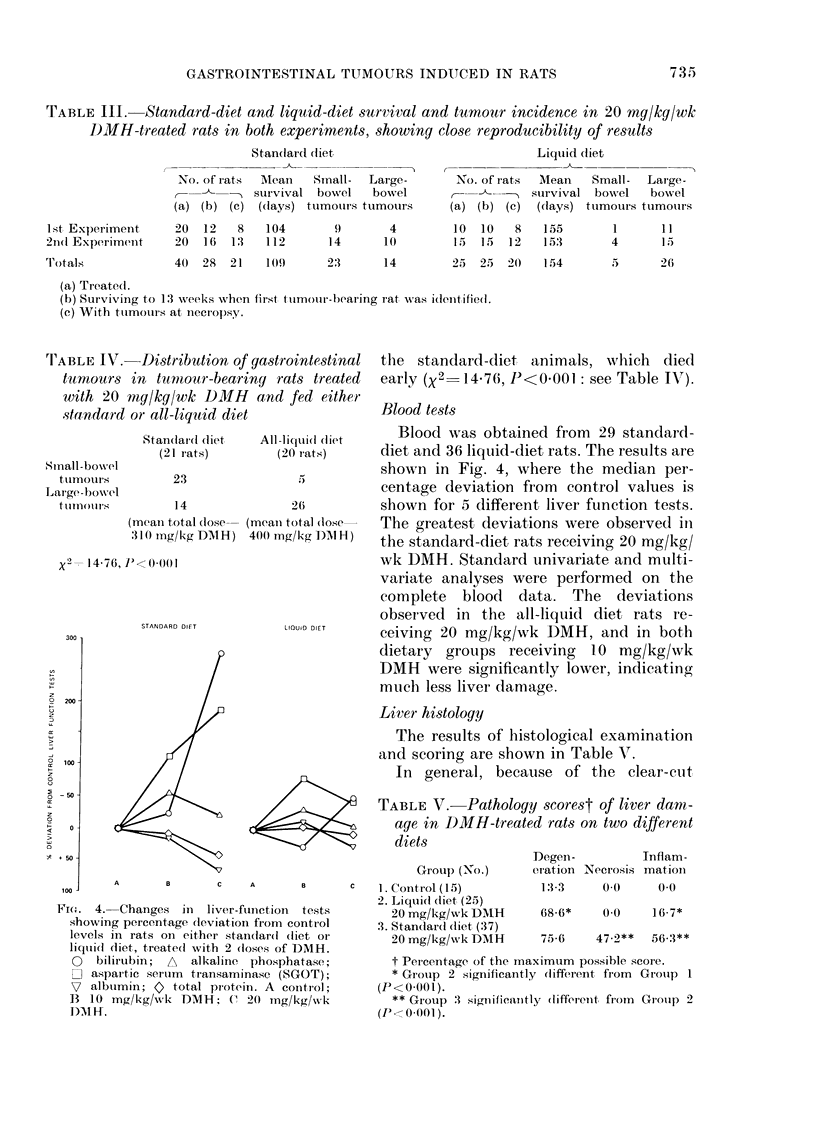

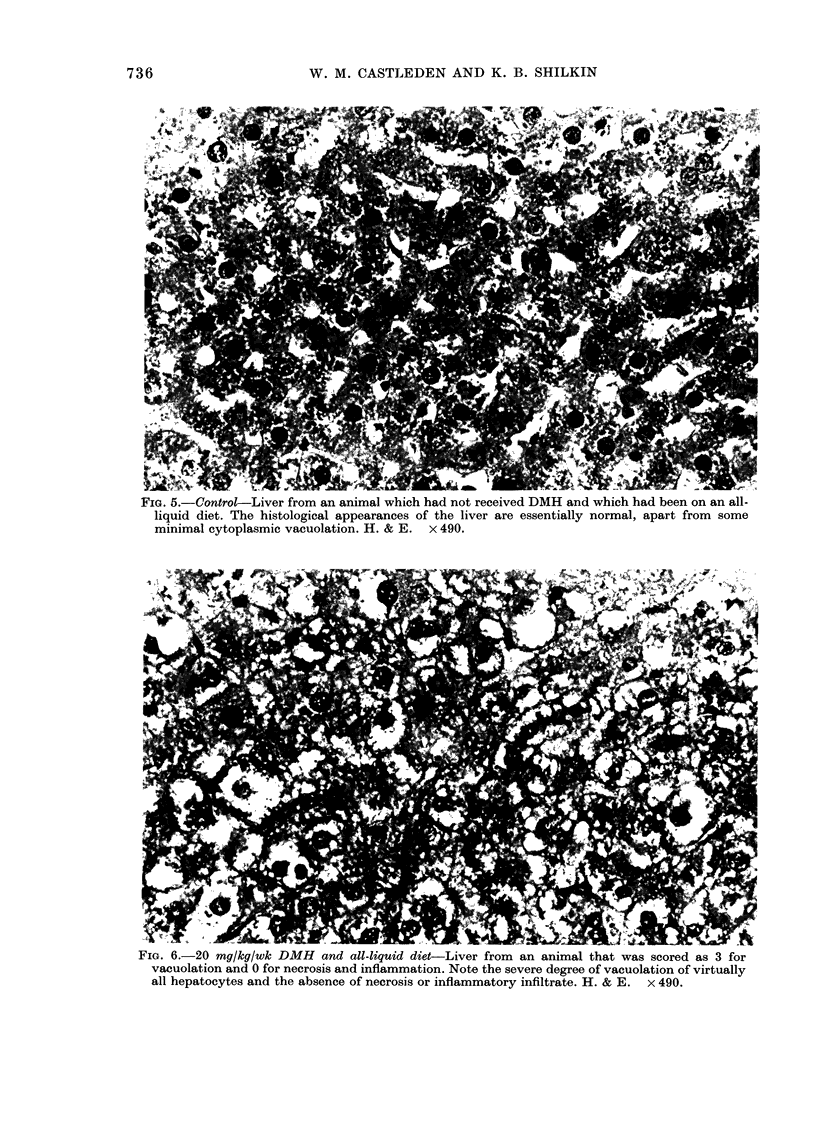

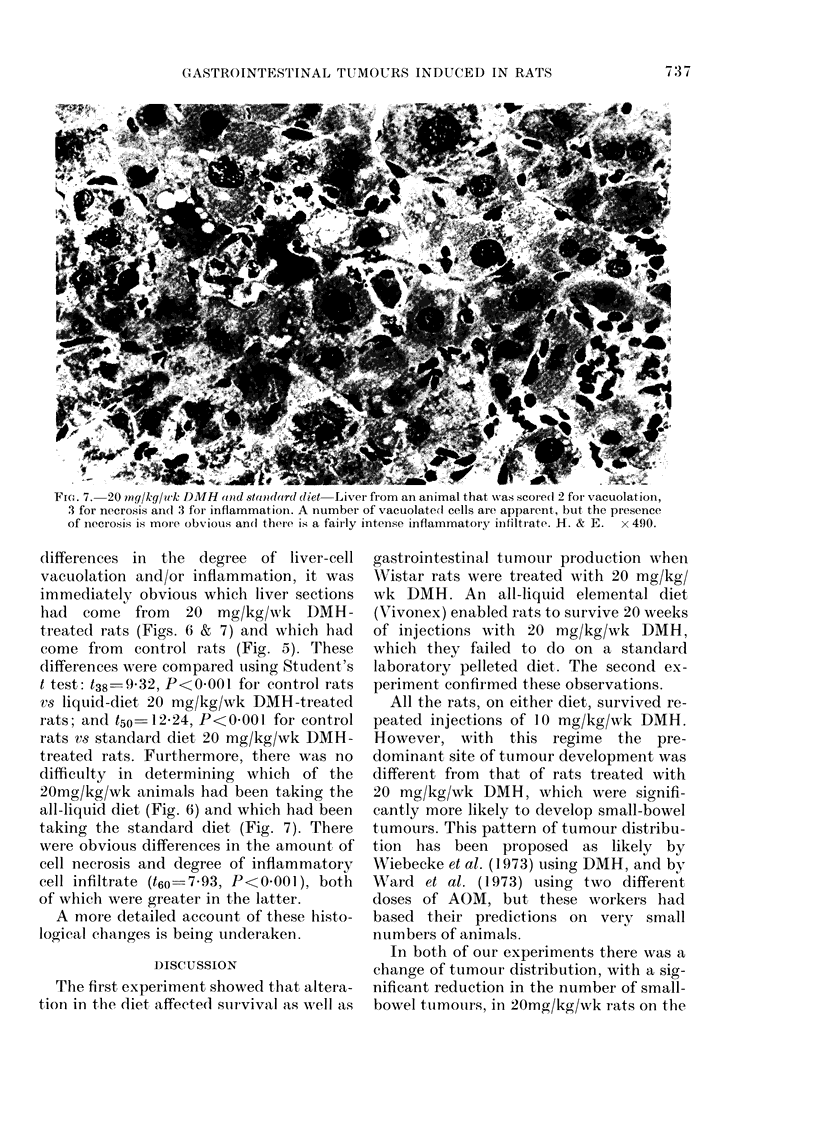

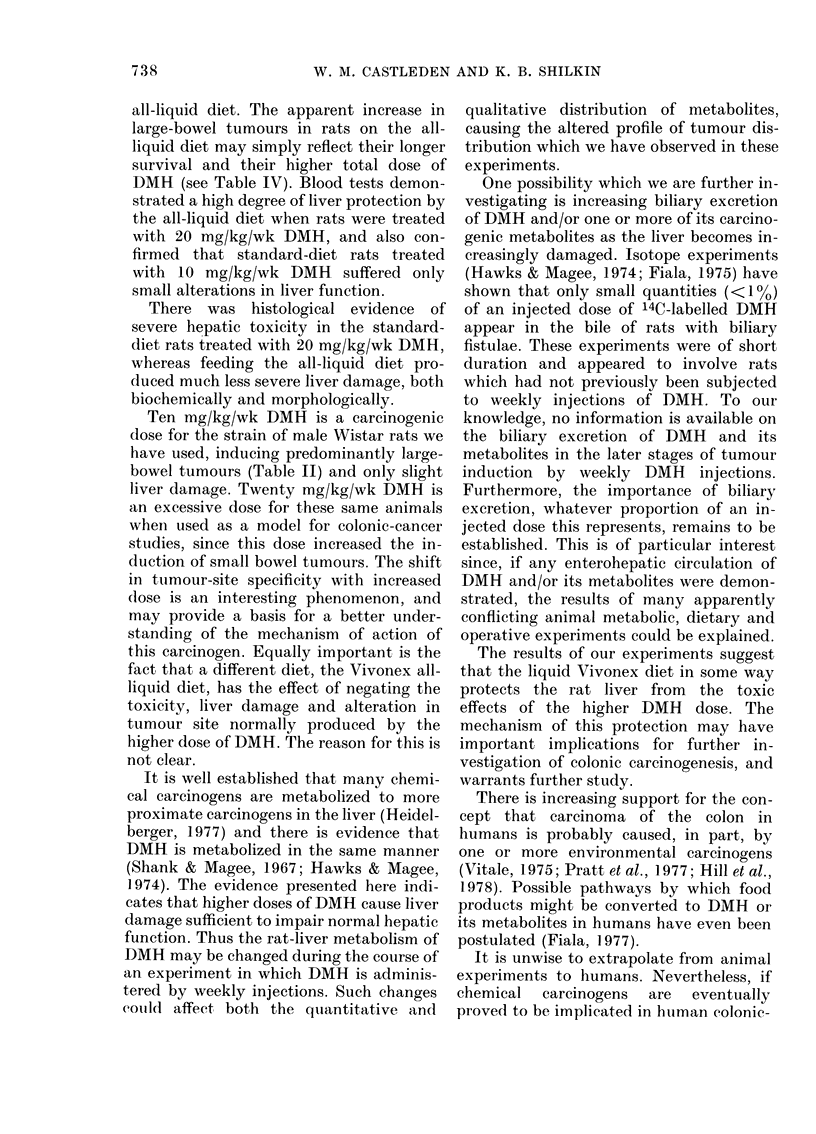

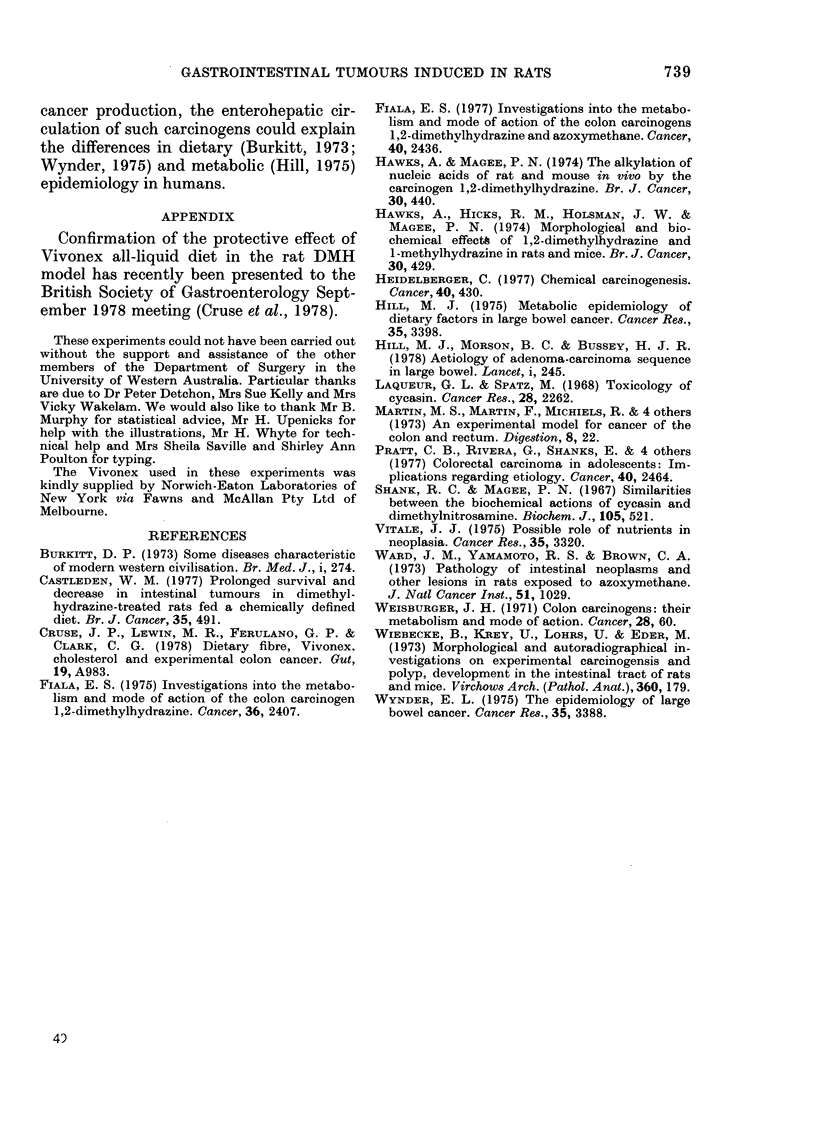


## References

[OCR_00828] Burkitt D. P. (1973). Some diseases characteristic of modern Western civilization.. Br Med J.

[OCR_00831] Castleden W. M. (1977). Prolonged survival and decrease in intestinal tumours in dimethylhydrazine-treated rats fed a chemically defined diet.. Br J Cancer.

[OCR_00848] Fiala E. S. (1977). Investigations into the metabolism and mode of action of the colon carcinogens 1,2-dimethylhydrazine and azoxymethane.. Cancer.

[OCR_00843] Fiala E. (1975). Investigations into the metabolism and mode of action of the colon carcinogen 1, 2-dimethylhydrazine.. Cancer.

[OCR_00860] Hawks A., Hicks R. M., Holsman J. W., Magee P. N. (1974). Morphological and biochemical effects of 1,2-dimethylhydrazine and 1-methylhydrazine in rats and mice.. Br J Cancer.

[OCR_00854] Hawks A., Magee P. N. (1974). The alkylation of nucleic acids of rat and mouse in vivo by the carcinogen 1,2-dimethylhydrazine.. Br J Cancer.

[OCR_00867] Heidelberger C. (1977). Chemical carcinogenesis.. Cancer.

[OCR_00871] Hill M. J. (1975). Metabolic epidemiology of dietary factors in large bowel cancer.. Cancer Res.

[OCR_00876] Hill M. J., Morson B. C., Bussey H. J. (1978). Aetiology of adenoma--carcinoma sequence in large bowel.. Lancet.

[OCR_00881] Laqueur G. L., Spatz M. (1968). Toxicology of cycasin.. Cancer Res.

[OCR_00890] Pratt C. B., Rivera G., Shanks E., Johnson W. W., Howarth C., Terrell W., Kumar A. P. (1977). Colorectal carcinoma in adolescents implications regarding etiology.. Cancer.

[OCR_00895] Shank R. C., Magee P. N. (1967). Similarities between the biochemical actions of cycasin and dimethylnitrosamine.. Biochem J.

[OCR_00900] Vitale J. J. (1975). Possible role of nutrients in neoplasia.. Cancer Res.

[OCR_00904] Ward J. M., Yamamoto R. S., Brown C. A. (1973). Pathology of intestinal neoplasms and other lesions in rats exposed to azoxymethane.. J Natl Cancer Inst.

[OCR_00910] Weisburger J. H. (1971). Colon carcinogens: their metabolism and mode of action.. Cancer.

[OCR_00914] Wiebecke B., Krey U., Löhrs U., Eder M. (1973). Morphological and autoradiographical investigations on experimental carcinogenesis and polyp development in the intestinal tract of rats and mice.. Virchows Arch A Pathol Pathol Anat.

[OCR_00920] Wynder E. L. (1975). The epidemiology of large bowel cancer.. Cancer Res.

